# Phenylalanine Ammonia-Lyase as a Key Enzyme in Tea Plant Resistance to Herbivory

**DOI:** 10.3390/ijms27010113

**Published:** 2025-12-22

**Authors:** Ran Wang, Zhichao Chai, Yongchen Yu, Xiaona Qian, Jia Wang, Xiaoling Sun, Xin Zhang

**Affiliations:** 1 Key Laboratory of Biology, Genetics and Breeding of Special Economic Animals and Plants, Ministry of Agriculture and Rural Affairs, National Center for Tea Plant Improvement, Tea Research Institute, Chinese Academy of Agricultural Sciences, Hangzhou 310008, China; bjsale40@transgen.com (R.W.); 17814680074@163.com (Z.C.); yuyongchen@tricaas.com (Y.Y.); qxnqxn0412@163.com (X.Q.); lvhenglei@weichai.com (J.W.); 2 State Key Laboratory of Tea Plant Germplasm Innovation and Resource Utilization, Hangzhou 310008, China

**Keywords:** *Camellia sinensis*, phenylalanine ammonia-lyase, herbivore resistance, catechins, heterologous expression

## Abstract

The tea plant (*Camellia sinensis*) employs inducible chemical defenses against insect herbivores, yet the role of phenylalanine ammonia-lyase (PAL) in this process remains inadequately characterized. This study demonstrates that PAL is essential for tea plant’s direct resistance against the tea geometrid (*Ectropis grisescens* Warren). Inhibition of PAL activity using 2-Aminoindan-2-phosphonic acid significantly reduced catechins accumulation and promoted larval growth of *E. grisescens*. Compared to mechanical wounding alone, simulated herbivory feeding (mechanical wounding plus oral secretions) induced higher PAL activity and more pronounced upregulation of *CsPAL* genes. This response specifically highlighted *CsPALb*, *CsPALd*, and *CsPALe* as core, herbivore-responsive members. Transient silencing of *CsPALb* in tea leaves led to a significant reduction in the levels of catechin (-)-epigallocatechin and epigallocatechin gallate. Moreover, heterologous overexpression of *CsPALb* and *CsPALd* in tobacco (*Nicotiana tabacum*) enhances resistance to *Spodoptera litura*. Our results indicate that PAL-mediated phenylpropanoid metabolism is not only critical for herbivore resistance of tea plant, but can also provide valuable gene resources for improving herbivore resistance in other plants.

## 1. Introduction

According to fossil records, arthropods originated in the mid-Carboniferous (~300 Ma), approximately 100 million years after the emergence of vascular plants [1]. In the long evolution process, herbivores always impose evolutionary pressures on plants. Plants deploy sophisticated transcriptional and metabolic reprogramming that drives the accumulation of amazing diversity of specialized metabolites upon herbivore infestation, which emphasizes the complexity of plant defense [2,3,4]. These metabolites, including green leaf volatiles, terpenoids (such as hemiterpenoids, monoterpenoids, sesquiterpenoids, diterpenoids), phenolic compounds (such as phenolic acids, flavonoids, quinones, lignans, stilbenes, and tannins), defensive proteins (such as protease inhibitors, lectins, chitinases, redox enzymes), and nitrogen-containing compounds (alkaloids, cyanogenic glycosides, and sulfur-containing glucosinolates), enable multi-layered defenses through direct and indirect mechanisms. Their production is regulated upstream by phytohormone-mediated signaling pathways to maximize plant fitness [5,6,7,8,9,10]. Among them, defensive proteins, phenolic and nitrogen-containing compounds are critical components of direct defense [11]. Notably, phenolic compounds represent the most salient anti-herbivore defense system in plants entire lifespan due to their unparalleled chemical diversity and multifunctional roles (such as cell wall reinforcement, direct toxicity and disruption of insect hormonal regulation). These compounds originate from shikimic acid–phenylpropanoid pathway in vascular plants [12].

In vascular plants, the shikimic acid pathway is a key metabolic route that converts phosphoenolpyruvate (PEP) and erythrose-4-phosphate into aromatic amino acids, such as phenylalanine, tyrosine, tryptophan via chorismite [12]. Phenylalanine serves as the primary substrate for the phenylpropanoid pathway [13]. The initial and rate-limiting step in phenylpropanoid pathway is catalyzed by phenylalanine ammonia-lyase (PAL, EC 4.3.1.24), which mediates the non-oxidative deamination of phenylalanine to produce *trans*-cinnamic acid and ammonia [14]. The deamination reaction represents the committed entry point into phenylpropanoid metabolism, ultimately leading to the biosynthesis of diverse phenolic compounds and salicylic acid, both of which play critical roles in plant physiology and stress responses [14,15,16,17]. PAL induction and activity represent a stress-responsive mechanism triggered by both biotic and abiotic stressors [18,19,20,21]. PAL expression and activity in herbaceous plants positively regulate resistance against both pathogens and herbivores through multilayered mechanisms [22,23,24,25]. For example, PAL activity contributes to the resistance of black rice (a black colour mutation of *Oryza sativa*) against bacterial leaf blight *Xanthomonas oryzae* pv. *oryzae* (*Xoo*) [25]; tobacco (*Nicotiana tabacum* L. cv. Xanthinc) with epigenetically suppressed PAL expression fails to develop systemic acquired resistance to tobacco mosaic virus [22]; knockdown of *OsPALs* in rice plant compromises brown planthopper (*Nilaparvata lugens*) resistance, while *OsPAL8* overexpression enhances resistance by regulating salicylic acid and lignin biosynthesis [26]; and *MePAL6*-mediated lignin accumulation confers resistance of cassava (*Manihot esculenta*) to two-spotted spider mite (*Tetranychus urticae*) [27]. Although multiple downstream genes of PAL have been identified to mediate herbivore resistance in woody perennials, the specific role of phenylalanine ammonia-lyase (PAL) remains poorly understood [28,29,30].

The tea plant (*Camellia sinensis*) is a woody perennial cash crop, valued for its tender buds and leaves which are processed into one of the world’s most consumed non-alcoholic beverages [31]. In the field, tea plants face diverse herbivores that severely compromise yield and quality. Among these pests, the tea geometrid sibling species, *Ectropis obliqua* Prout and *E. grisescens* Warren, are the most prevalent herbivores in Chinese tea plantations [32,33]. Upon recognizing chemical elicitors from tea geometrids, the tea plant activates a direct defense resistance mediated by a diversity of phenolic compounds, including catechins, naringenin, polyphenol oxidase, and quercetin-3-O-glycosides. The defense process is collectively modulated by jasmonic acid, auxin, and ethylene mediated signaling pathways [6,34,35]. Although the genes encoding PAL and downstream phenylpropanoid pathway enzymes—including 4-coumarate: CoA ligase, flavonoid 3′5′-hydroxylase, and chalcone synthase—have been confirmed to be upregulated by tea geometrid infestation and jasmonates application [6], mechanistic evidence for how PAL mediates herbivore resistance in tea plants by regulating phenolic compound biosynthesis is still lacking.

In tea plants, six PAL genes (*CsPALs*) have been verified to exhibit substrate specificity for L-phenylalanine but display divergent phylogenetic relationships, tissue-/induced-expression patterns, and enzymatic kinetics [36]. This study employed a randomized complete block design, with all biological replicates conducted under spatiotemporally consistent conditions and with treatments as the sole variable. First, the PAL inhibitor—2-Aminoindan-2-phosphonic acid (AIP) is used to verify that PAL enzyme positively regulates tea plant resistance to *E. grisescens* via modulating the accumulation of catechins. Next, the oral secretions of *E. grisescens* caterpillars upregulates PAL enzyme activities and *CsPALs* expression levels. Finally, transient silence of *CsPALb* reduces the accumulation of catechins in tea plants remarkably, and the heterologous expression of *CsPALb* and *CsPALd* increases tobacco (*Nicotiana tabacum*) resistance to *Spodoptera litura* caterpillars. This study provides evidence that PAL plays a critical role in tea plant herbivore resistance, with a specific focus on dissecting the divergent function of two key isoforms, *CsPALb* and *CsPALd*. These findings not only elucidate the molecular mechanisms underlying herbivore resistance but also provide valuable candidate gene resources for insect-resistant breeding.

## 2. Results

### 2.1. PAL Enhances Tea Plant Resistance to E. grisescens by Regulating Catechins Accumulation

**Figure 1:**
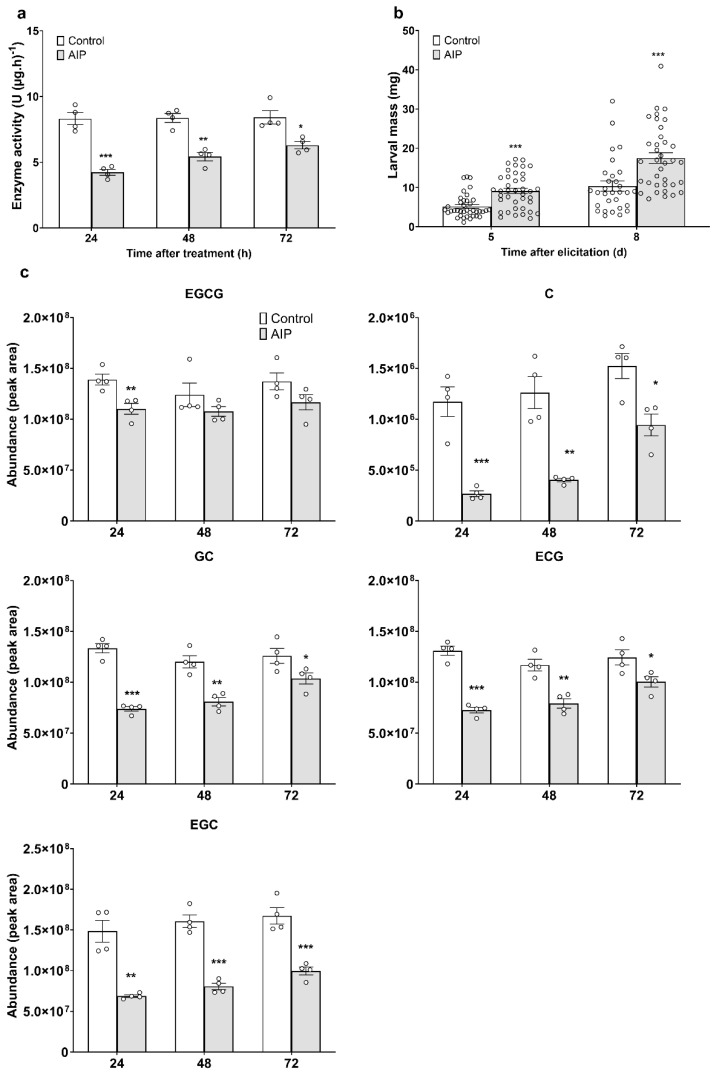
Phenylalanine ammonia-lyase (PAL) positively regulates tea plant resistance to *E*. *grisescens* via modulating the accumulation of catechins. (**a**,**b**) The treatment of PAL inhibitor-2-Aminoindan-2-phosphonic acid (AIP) inhibits PAL activity (**a**) and improves the growth of *E*. *grisescens* (**b**). (**c**) The application of AIP inhibits the accumulation of catechins. Asterisks indicate significant differences between two treatments (* *p* < 0.05, ** *p* < 0.01, *** *p* < 0.001, Student’s *t*-test).

To investigate the function of PAL, the PAL inhibitor AIP was employed to suppress PAL activity. The results showed that AIP application significantly inhibited PAL activity in tea plants, with reductions of 49%, 34%, and 25% observed at 24, 48, and 72 h, respectively (Figure 1a). This sustained suppression demonstrates that AIP effectively suppresses PAL activity in tea plants. Meanwhile, the weight gain of *E. grisescens* larvae fed on AIP-treated leaves was significantly higher compared to those fed on control leaves, with increases of 77% on day 5 and 69% on day 8, respectively (Figure 1b). Furthermore, chemical analysis revealed that AIP treatment significantly reduced the levels of catechins compared to the control. The affected compounds included (-)-epigallocatechin gallate (EGCG), (+)-catechin (C), (-)-gallocatechin (GC), (-)-epicatechin gallate (ECG), and (-)-epigallocatechin (EGC). Marked reductions were observed at 24, 48, and 72 h: EGCG showed declines of 21%, 13%, and 15%; C, 77%, 68%, and 38%; GC, 45%, 33%, and 18%; ECG, 45%, 32%, and 19%; and EGC, 54%, 50%, and 41% (Figure 1c).These results suggest that AIP inhibits the accumulation of defensive catechins by suppressing PAL activity, thereby enhancing the larval growth rate of *E. grisescens* larvae.

### 2.2. CsPALb and CsPALd Are Key Candidates in Tea Plant Resistance to E. grisescens

**Figure 2:**
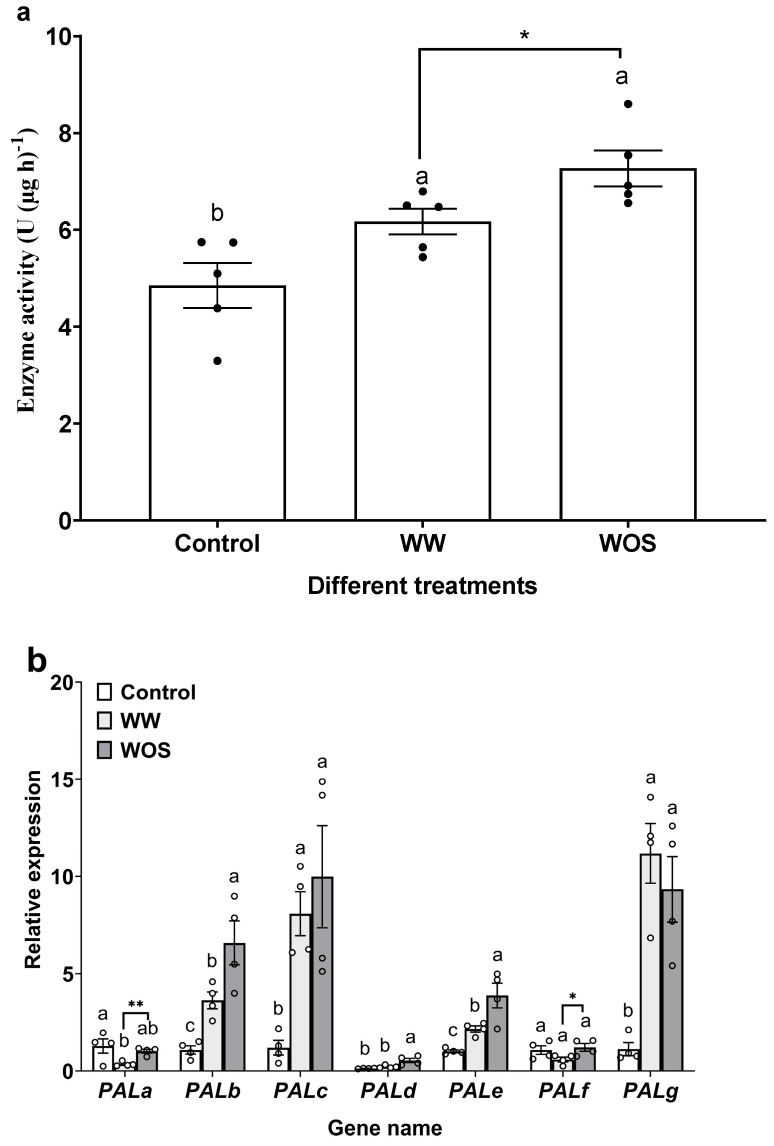
Simulated feeding of *Ectropis grisescens* elicited PAL activity (**a**) and *CsPALs* expression levels (**b**). Control: intact tea plant; WW: healthy tea plant treated with mechanical wounding plus deionized water; WOS: healthy tea plant treated with mechanical wounding plus oral secretion. Different letters indicate significant differences among treatments (*p* < 0.05, Tukey’s honest significant difference post hoc test, *n* = 4). Statistical significance between WOS and WW was assessed by Student’s *t*-test, with significant differences denoted by an asterisk (* *p* < 0.05, ** *p* < 0.01, *n* = 4).

We further analyzed the expression patterns of different PAL family members in response to mechanical wounding and simulated herbivory using qRT-PCR. Compared to control, both mechanical wounding plus water (WW) and wounding plus oral secretion (WOS) significantly enhanced PAL activity. Compared to WW, WOS significantly enhanced PAL activity, with the induction by WOS being 1.18-fold higher than that by WW (Figure 2a). Gene expression profiling showed that both treatments up-regulated multiple *CsPAL* genes, with WOS inducing a stronger response. A significantly stronger induction of *CsPALb*, *CsPALd*, and *CsPALe* was observed with WOS treatment compared to WW, with increases of 1.81-, 2.52-, and 1.79-fold, respectively (Figure 2b). Among the three elicited PAL genes, *CsPALd* was specifically induced by WOS coupled with its low basal expression, whereas both *CsPALb* and *CsPALe* were induced by either WW or WOS. However, the induction fold of *CsPALb* by WOS was higher. Consequently, our study focused on *CsPALb* and *CsPALd* to dissect their distinct roles in the regulation of anti-herbivore defenses.

### 2.3. Differential Effects of CsPALb and CsPALd Silencing on Catechins Metabolism

**Figure 3:**
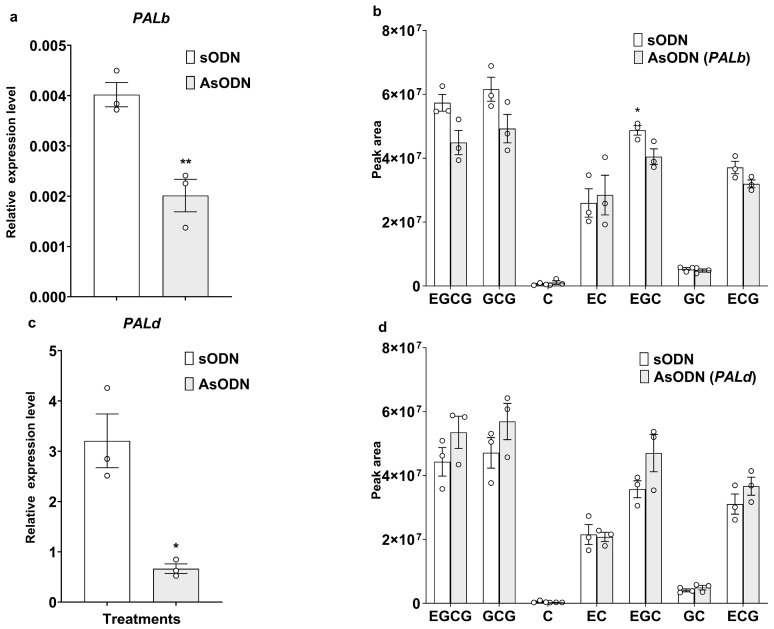
Transient silence of *CsPALs* reduce the expression levels (**a**,**c**) and the accumulation of catechins in tea plants (**b**,**d**). Asterisk indicates significant differences between two treatments (* *p* < 0.05, ** *p* < 0.01, Student’s *t*-test).

To dissect their specific roles, we individually silenced *CsPALb* and *CsPALd* in tea plants using antisense oligonucleotide (AsODN) technology. Expression of *CsPALb* and *CsPALd* were successfully silenced and the transcript levels downgraded by approximately 50% and 79%, respectively (Figure 3a,c). Catechins analysis revealed that silencing *CsPALb* caused a significant reduction in EGC content, with its accumulation decreasing to approximately 83% of the control level. Similarly, the levels of EGCG and ECG also showed marked reductions, reaching about 78.3% and 86.2% of the control, respectively. Surprisingly, silencing *CsPALd* had no significant effect on the measured catechins levels (Figure 3b,d).

### 2.4. CsPALb and CsPALd Overexpression Enhances Tobacco Resistance to Spodoptera Litura

**Figure 4:**
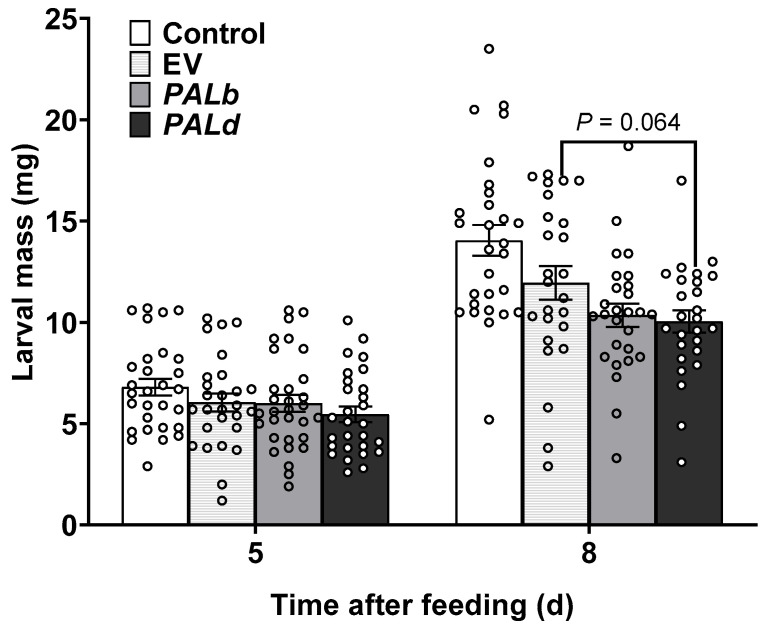
The heterologous overexpression of *CsPALs* increases the tobacco (*Nicotiana tabacum*) resistance to *Spodoptera litura* caterpillars. Statistical significance between EV (empty vector) and *CsPALd-OE* was assessed by Student’s *t*-test (*n* > 24).

We further investigated the defensive potential of these genes through transient overexpression in tobacco. Leaves expressing *CsPALb* or *CsPALd* were fed to *Spodoptera litura* larvae. After 5 days of feeding, larvae fed with *CsPALb-OE* and *CsPALd-OE* leaves exhibited body weight reductions of 10.91% and 19.72%, respectively, relative to the control, and 0.69% and 9.65% relative to the EV group. By day 8, this growth-suppressive effect was more pronounced, with weight reductions reaching 26.28% for *CsPALb-OE* and 28.42% for *CsPALd-OE* compared to the control, and 13.30% and 15.83% compared to the EV group. These results indicate that overexpression of both *CsPALb* and *CsPALd* in tobacco confers a growth-inhibitory effect against *S. litura* larvae (Figure 4).

## 3. Discussion

PAL serves as a pivotal entry-point enzyme in the phenylpropanoid pathway, orchestrating the biosynthesis of a vast array of phenolic compounds that are central to plant defense against both pathogens and herbivores. Its conserved role in enhancing disease resistance has been well-established across several herbaceous species. However, its specific function and regulatory mechanisms in the anti-herbivore defense of woody perennials, particularly in an economically important crop like tea plants, remain less elucidated.

The present study investigated the role of PALs in the defense mechanisms of tea plants against tea geometrids. Sustained inhibition of PAL activity using the specific inhibitor AIP significantly reduced several defensive catechins levels (e.g., EGCG, C, GC, ECG, and EGC) in tea leaves (Figure 1c). Concomitantly, *E. grisescens* larvae feeding on AIP treated plants exhibited greater weight gain compared to those feeding on the controls (Figure 1b), indicating that PAL-mediated catechins biosynthesis is critical for anti-herbivore defense in tea plants. These findings align with reports on herbaceous plants, where PAL positively regulates systemic resistance in tobacco [22] and confers resistance to brown planthopper in rice [37]. Thus, this study extends the functional evidence of PALs in anti-herbivore defense from herbaceous plants to the woody perennial, suggesting a conservation mechanism across different plant lineages. Notably, the PAL gene family in tea plants comprises six members (*CsPALa*-*CsPALf*) that have undergone functional diversification, with distinct expression patterns and kinetic properties [36]. This complexity in a woody perennial may underpin a more nuanced regulatory network for phenolic compound biosynthesis in response to herbivory compared to annual herbs.

Simulated herbivory (WOS) significantly induced higher PAL enzyme activity than mechanical wounding alone, suggesting that specific elicitors present in the oral secretions amplify the plant’s defense responses. Compared to mechanical wounding alone, the expressions of *CsPALb* and *CsPALd* were specifically and strongly induced under WOS (Figure 2b). Previous studies have shown that *CsPAL* genes display tissue-specific and stimulus-responsive expression patterns. For instance, *CsPALb* is highly inducible by MeJA, whereas *CsPALe* and *CsPALf* are predominantly expressed in semi-lignified xylem [36]. Our findings demonstrate that tea plants preferentially regulate specific family members (e.g., *CsPALb* and *CsPALd*) upon insect herbivory to achieve a finely tuned defense response. Such functional diversification among PAL family members under biotic stress appears to be a common phenomenon in plants, as evidenced by the differential expression and functional specialization of the *AtPALs* in Arabidopsis and the *OsPALs* in rice during pathogen resistance [38]. Furthermore, genome-wide analyses in plants indicate that PAL genes are highly conserved but have evolved through lineage-specific duplications, contributing to functional diversity in stress responses [28]. The specific induction of *CsPALb*/*d* by herbivory in tea adds a layer to understanding how woody plants leverage gene family expansion for ecological adaptation.

To functionally characterize CsPAL isoforms in herbivore defense, gene silencing in tea and heterologous overexpression in tobacco were used to obtain preliminary evidence. Silencing *CsPALb* specifically reduced EGC, ECG and EGCG accumulation (Figure 3b), providing direct involvement in the biosynthesis of these defensive catechins. This finding not only confirms the role of PAL activity in catechin-based defense (Figure 1) but further identifies *CsPALb* as a contributing member to anti-herbivore activity. In contrast, silencing *CsPALd* did not result in decrease in any measured catechin component (Figure 3d), yet heterologous overexpression in tobacco showed a non-significant but notable trend toward reducing *S. litura* larval growth, an effect stronger than that of *CsPALb* overexpression (Figure 4). This highlights a functional divergence between *CsPALb* and *CsPALd* and underscores the complexity of PAL-mediated defense. Functional redundancy among CsPAL isoforms (e.g., *CsPALb* or *CsPALe*) may compensate for *CsPALd* transient loss, a phenomenon observed in other woody species such as walnut (*Juglans regia* L.) [29,30]. Concurrently, the enzyme’s noted catalytic efficiency [36] might favor metabolic flux toward branches other than catechin biosynthesis, such as those yielding structural or otherwise unmeasured defensive compounds. Studies in plants like poplar and rice have shown that PAL genes influence both phenolic metabolites and lignin content, affecting herbivore resistance (e.g., *OsPAL4* in rice disease resistance). Silencing of *MePAL6* in cassava (*Manihot esculenta*) alters plant resistance to the two-spotted spider mite (*Tetranychus urticae*) by modulating lignin accumulation [27]. In rice, both knockout and overexpression of *OsPALs* exert crucial effects on lignin biosynthesis and plant resistance to the brown planthopper (*Nilaparvata lugens*) [39]. These initial observations highlight the need for further investigation using stable genetic mutants and untargeted metabolomics to clarify the functional specialization and regulatory networks of *CsPALb* and *CsPALd*. Additionally, exploring substrate specificity among CsPAL isoforms, such as their preference for L-Phe [36], could reveal unique aspects of defense metabolism in tea plants.

In summary, our study confirms the conserved function of PAL in plant defense and reveals the functional specialization of *CsPAL* genes in a woody perennial system. The differential roles of *CsPALb* and *CsPALd* highlight the regulatory complexity of phenylpropanoid metabolism, offering novel genetic and mechanistic insights for improving insect resistance in tea and other perennial crops.

## 4. Materials and Methods

### 4.1. Plants and Insects

Two-year-old *Camellia sinensis* cv. ‘Longjing 43’ were used in this study. The tea plants were planted individually in plastic pots (14 cm diameter and 15 cm high), irrigated once every other day, and fertilized with rapeseed cake once a month. Healthy tea plants with a uniform height were selected for all experiments, which were carried in a controlled climate room (26 ± 2 °C, 70–80% relative humidity (rh), and 12 h photophase). The leaf that was most apical and had just begun to unfold, though not yet fully expanded, was designated as leaf position one, with the remaining leaves numbered in descending order down the stem.

*Nicotiana tabacum* plants were cultivated in a growth chamber (25 °C with 60% relative humidity, 16 h light/8 h dark photoperiod) under a light intensity of 8000 lx. Plants used in the experiment were at a vigorous growth stage, approximately one month old, and had not yet initiated flowering.

*Ectropis grisescens* larvae were originally collected from the experimental plantation of Tea Research Institute, Chinese Academy of Agricultural Sciences, Hangzhou, China, and reared with fresh tea shoots in a controlled climate room (26 ± 2 °C, relative humidity 70 ± 5%, 12 h light/12 h dark photoperiod). After one generation, three-day-old caterpillars were selected for the bioassay. Oral secretions (OS) were collected from fourth-instar caterpillars with a P200 Pipetteman, in accordance with the established method [6]. OS were then homogenized, aliquoted into 200 µL centrifuge tubes, and stored at −80 °C until use.

The *Spodoptera litura* larvae (third-instar) used in this study were obtained from a laboratory colony established from specimens collected in a Zhejiang Province cornfield and maintained by Prof. Yaobin Lu’s group at the Zhejiang Academy of Agricultural Sciences. The larvae were reared in insect rearing cages (60 cm × 60 cm × 60 cm) under controlled conditions: 25 ± 2 °C, relative humidity 60–80%, and a photoperiod of 14 h of light per day. Following one generation of rearing, three-day-old caterpillars were utilized for the bioassay.

### 4.2. Herbivore Performance Bioassays

Bioassays were conducted separately on tea and tobacco plants. In the bioassay, the second tender leaf of tea plants and the third fully expanded leaf of tobacco plants (from the top) were used. Each leaf was enclosed with a fine-mesh sleeve and then introduced with one 3-day-old caterpillar of *E. grisescens* (starved for 4 h) or *S. litura*, respectively. The larvae were moved to an adjacent, tender intact leaf (on the same branch for tea; same plant for tobacco) when the initial leaf was nearly half-consumed, to ensure a continuous food supply. Larval mass was measured using an electronic balance (precision: 0.001 g) at 5- and 8 days post introduction.

### 4.3. Plants Treatment

AIP Treatment. 2-Aminoindan-2-phosphonic acid (AIP), an efficient PAL inhibitor, irreversibly inhibits PAL activity by covalently binding to its active site as a transition-state analog [40]. Based on the AIP application concentration established in *Petunia hybrida* [41], AIP (Sigma, St. Louis, MO, USA) was prepared as a 0.5 mM solution using ddH_2_O. A volume of 5 mL of 0.5 mM AIP solution was uniformly sprayed on the whole tea plants with a 10 mL sterilized spray bottle; this treatment was repeated once after 24 h, and the leaf surface was air-dried following each spray. Tea plants sprayed only with ddH_2_O served as the control. Subsequently, part of the control and AIP-treated tea plants were used for the determination of total PAL enzyme activity and catechins content with four biological replicates per group. Leaf samples were collected at 24, 48, and 72 h post the final treatment. The remaining tea plants were used for the bioassay.

Oral Secretion Treatment. Mechanical wounding plus oral secretion (WOS) were used to simulate herbivore feeding, with mechanical wounding plus deionized water (WW) serving as the control. On the second leaf of tea plant, two parallel incisions were made on each side of the midvein using a pattern wheel, and 10 μL of either deionized water or OS was applied to each wound. Samples of the intact control, WW and WOS for determining the total PAL enzyme activity were harvested at 24 h post treatment, with five biological replicates per group. Samples for gene expression analysis were harvested at 12 h post treatment.

### 4.4. Measurement of Phenylalanine Ammonia-Lyase Activity

Total PAL enzyme activity was measured according to [42] with minor modifications. Briefly, 0.1 g of powdered tissue sample was homogenized in 1 mL of extraction buffer. The buffer consisted of 100 mmol/L borate buffer (pH 8.8) containing 4% (*w*/*v*) PVP, 78 mmol/L EDTA, and 14.3 mmol/L β-mercaptoethanol. A small amount of quartz sand was added, and the homogenization was conducted on an ice bath. Then the sample was transferred to a centrifuge tube and centrifuged at 10,000 rpm for 15 min at 4 °C. The supernatant, designated as the crude enzyme extract, was collected and stored at −20 °C for subsequent analysis. For the enzyme activity assay, 20 µL of 0.02 mol/L L-phenylalanine solution and 120 µL of 0.05 mol/L borate buffer were aliquoted into a well of a 96-well quartz microplate. Then, 20 µL of the crude enzyme extract was added. The reaction mixture was incubated at 37 °C for 60 min and then terminated by adding 4 µL of 6 mol/L HCl. After standing for 5 min, the absorbance at OD_290_ was measured. We determined the protein concentration by the Bradford method with Coomassie Brilliant Blue G-250, using bovine serum albumin as the standard and measuring the absorbance at 595 nm. The enzyme activity was calculated and expressed in units of 0.01 ΔA_290_ per hour per microgram of protein (0.01 ΔA_290_·h^−1^·μg^−1^).

### 4.5. Catechins Analysis

The catechin contents in the control, AIP-treated, and AsODNs-treated tea plants were measured, with four or three biological replicates per group, respectively. Catechins were quantified as described in the International Standard method (ISO 14502-2:2005) [43] with minor modifications. Briefly, 100 mg of powdered leaf sample was extracted with 1 mL of 70% (*v*/*v*) aqueous methanol. Extracts were subsequently analyzed using high-performance liquid chromatography (HPLC). Chromatographic separation was performed on a Waters e2695 HPLC system (Waters Corp., Milford, MA, USA) fitted with a reversed-phase C_12_ analytical column (4 μm, 250 mm × 4.6 mm (i.d.); Phenomenex) protected by a corresponding C_12_ guard column. Detection was carried out using a diode array detector set at 280 nm. Identification of each compound was achieved by matching retention time with reference standard. Quantification was performed via the external standard method. The relative abundance of each catechin component was determined by the peak area normalization method, and is expressed as the percentage of its individual peak area relative to the total peak area of all catechins. All reference standards were procured from Sigma-Aldrich (St. Louis, MO, USA).

### 4.6. RNA Extraction and Quantitative Real-Time PCR Analysis

The TeaPGDB (http://eplant.njau.edu.cn/tea/index.html, accessed on 16 December 2022) was used to access the ‘Shuchazao’ genome database. Seven *PAL* gene sequences of the tea tree (*CsPALa-g* gene numbers: CSS0041448.1, CSS0048281.1, CSS0018870.1, CSS0001442.1, CSS0034802.1, CSS0010367.1, CSS0021474.1) were downloaded. Primers were designed using Primer Premier 5. Total RNA was isolated using the TRIzol™ reagent (TIANGEN, Beijing, China) following the manufacturer’s protocol. First-strand cDNA synthesis was carried out with the PrimerScript™ RT Reagent Kit (TaKaRa, Dalian, China). qRT-PCR assays were conducted on a LightCycler^®^ 480 instrument (Roche, Basel, Switzerland) using SYBR Green I Master Mix (Roche, Basel, Switzerland). The *CsGAPDH* was used as the reference gene, and relative expressions were calculated using the 2^−ΔΔCt^ method. The sequences of the specific primers used are described in Supplementary Table S1.

### 4.7. AsODN-Mediated Knockdown of CsPALb/d Expression in Camellia sinensis

The candidate antisense oligonucleotides (AsODNs) of *CsPALb*/*d* were designed and selected by Soligo software (version 2.2, https://sfold.wadsworth.org/cgi-bin/index.pl, accessed on 20 January 2024). The sense oligonucleotides (sODN) and antisense oligonucleotide (AsODN) were listed in Supplementary Table S2, and they were synthesized by Tsingke Biotechnology Co., Ltd. (Hefei, China). One milliliter of 20 μM AsODN-CsPALb/d solution was injected into the abaxial side of young tea leaves using a syringe, with a sense oligonucleotide (sODN) solution serving as the control. Gene expression analysis conducted 24 h post-injection, and catechins were detected 48 h after injection. Each experiment included at least three independent biological replicates.

### 4.8. Construction of CsPALb/d-GFP and Transient Expression in N. tabacum

The full-length coding sequences of *CsPALb* and *CsPALd* were amplified from tea leaf cDNA. Each PCR product was subsequently cloned into a GFP tag expression vector EV (35S promoter) using an In_Fusion HD Cloning kit (Takara, Tokyo, Japan), yielding the constructs *35Spro:CsPALb-GFP* and *35Spro:CsPALd-GFP*, respectively. All primers used for construction are listed in Supplementary Table S3. The constructions were then introduced into Agrobacterium strain GV3101. Transient expression in *N. tabacum* was performed as previously described [44]. Following infiltration, plants were maintained under appropriate conditions for at least 48 h prior to microscopic observation and herbivore performance bioassay.

### 4.9. Statistical Analysis

All data were analyzed using Statistica (version 9.4, SAS Institute Inc., Cary, NC, USA). Differences between the control and AIP-treated groups, as well as between sODN- and AsODN-injected tea leaves, were assessed using Student’s *t*-test. One-way ANOVA was used to assess differences among three or more groups, including *PALs* gene expression, larval mass of *Spodoptera litura*, and PAL enzyme activity in response to simulated herbivory. If the ANOVA analysis was significant (*p* < 0.05), Tukey’s honest significant difference (HSD) post hoc test was applied to compare differences among groups.

## Data Availability

All data needed to evaluate the conclusions in the paper are present in the paper and/or the Supplementary Materials.

## Supplementary Materials

The following supporting information can be downloaded at https://www.mdpi.com/article/10.3390/ijms27010113/s1.
