# Supplementary material for: Phenylalanine Ammonia-Lyase as a Key Enzyme in Tea Plant Resistance to Herbivory
Source: Int J Mol Sci. 2025 Dec 22;27(1):113. doi: 10.3390/ijms27010113 (PMC12785785; doi:10.3390/ijms27010113)
Supplement: Supplementary file 1 [file ijms-27-00113-s001.zip › ijms-3990166-supplementary.pdf]

International Journal of Molecular Sciences

Article

Phenylalanine Ammonia-Lyase as a Key Enzyme in Tea Plant Resistance to Herbivory

Ran Wang [1,2,†], Zhichao Chai [1,2,†], Yongchen Yu [1,2], Xiaona Qian [1,2], Jia Wang [1,2], Xiaoling Sun [1,2,*] and Xin Zhang [1,2,*]

[1] Key Laboratory of Biology, Genetics and Breeding of Special Economic Animals and Plants, Ministry of Agriculture and Rural Affairs, National Center for Tea Plant Improvement, Tea Research Institute, Chinese Academy of Agricultural Sciences, Hangzhou 310008, China; bjsale40@transgen.com (R.W.); 17814680074@163.com (Z.C.); yuyongchen@tricaas.com (Y.Y.); qxnqxn0412@163.com (X.Q.); lvhenglei@weichai.com (J.W.)

[2] State Key Laboratory of Tea Plant Germplasm Innovation and Resource Utilization, Hangzhou 310008, China

* Correspondence: xlsun1974@163.com or xlsun@tricaas.com (X.S.); xinzhang@tricaas.com (X.Z.)

† These authors contributed equally to this work.

Supplementary Table S1. qRT-PCR primers.

Gene	Primer (5′-3′)
qRT-PALa-F	TGACGTGGAAGTCAAGGTGG
qRT-PALa-R	CTCAAACCGGATGCCTGAGT
qRT-PALb-F	TCACTGTGCTACAAGGGCTG
qRT-PALb-R	TTGGTCCGGCCAGTTAAGAC
qRT-PALc-F	TGAGCACGCCTTGAAAAATGG
qRT-PALc-R	ATTTCCACTCTCAACCGCTCT
qRT-PALd-F	CACAATTCAAAGGCGGTCGG
qRT-PALd-R	ATAGAAGCCAAGCCGGAACC
qRT-PALe-F	AAGGCTACTCTGGCATTCGG
qRT-PALe-R	AAGGCCTCGGTTGCATTGAG
qRT-PALf-F	TGACGTGGAAGTCAAGGTGG
qRT-PALf-R	CTCAAACCGGATGCCTGAGT
qRT-PALg-F	AATGGCGACGCTGAGAAGAA
qRT-PALg-R	AGCGCCATTCCAACTCTTGA

Supplementary Table S2. Oligonucleotide silencing primers.

Gene	Primer (5′-3′)
Control	GGCGGCTAACGCTTCGA
PALb	ATTGCGTCCGCCAGAGAGAT
PALd	AACGCGCTTCGCTACTTGGC

Supplementary Table S3. Primers for homologous recombination.

Gene	Primer (5′-3′)
PALb-GFP-F	GAGCTCGGTACCCGGGGATCCGACTACAAGGACGACGATGACAAGATGGAGAATGCTAATGAG
PALb-GFP-R	CATGTCGACTCTAGAGGATCCACAAATTGGAAGAGGTGC
PALd-GFP-F	GAGCTCGGTACCCGGGGATCCGACTACAAGGACGACGATGACAAGATGGATAGTACCACCGCCATT
PALd-GFP-R	CATGTCGACTCTAGAGGATCCACAGATAGGAAGAGGAGC
PALb-GFP2300-F	GGACGAGCTGTACAAGGGATCCATGGAGAATGCTAATGAGAG
PALb-GFP2300-R	CAGGTCGACTCTAGAGGATCCACAAATTGGAAGAGGTGC
PALd-GFP2300-F	GGACGAGCTGTACAAGGGATCCATGGATAGTACCACCGCCATT
PALd-GFP2300-R	CTAACAAATTGGAAGAGGTGACAGATAGGAAGAGGAGC
